# Perioperative machine learning models with SHAP interpretation for predicting adverse outcomes in breast cancer surgery

**DOI:** 10.3389/fsurg.2026.1763803

**Published:** 2026-04-29

**Authors:** Yongde Yang, Jiawei Xu, Rong Liao, Yanlin Zhou

**Affiliations:** 1 Breast Center, Chongqing University Three Gorges Hospital, Wanzhou, Chongqing, China; 2 Department of General Surgery, the First Affiliated Hospital of Changsha Medical University, Changsha, Hunan, China; 3 Health Management Center, Chongqing University Three Gorges Hospital, Wanzhou, Chongqing, China

**Keywords:** breast cancer, external validation, machine learning, postoperative prognosis prediction, SHAP analysis

## Abstract

**Objective:**

To investigate the clinical value of a machine learning model constructed using perioperative data for predicting adverse postoperative outcomes in patients undergoing breast cancer surgery, and to identify key decision factors through SHAP interpretability analysis.

**Methods:**

Perioperative core indicators and follow-up data from 643 treatment-naïve patients with breast cancer who underwent surgical treatment were retrospectively collected, including 443 cases in the modeling set and 200 cases in the external validation set, derived from two independent medical centers. The modeling set was stratified and split into training and internal validation sets in 7:3 ratio. After screening key variables using univariate analysis in the training set, five predictive models for postoperative adverse prognosis were developed based on Extreme Gradient Boosting (XGBoost), Random Forest (RF), Gradient Boosting Machine (GBM), Support Vector Machine (SVM), and Logistic Regression (LR) algorithms. The model performance was evaluated using the area under the receiver operating characteristic curve (AUC), calibration curves (CC), and decision curve analysis (DCA) in both the internal and external validation sets, and the feature contributions of the optimal model were interpreted using the Shapley Additive exPlanations (SHAP) method.

**Results:**

The predictive model for postoperative adverse prognosis constructed using the XGBoost algorithm demonstrated optimal performance, showing strong discriminatory ability in both the internal (AUC = 0.840) and external (AUC = 0.780) validation sets. In the external validation set, its specificity (0.881) and F1 score (0.514) were higher than those of the other models. In addition, calibration analysis indicated good agreement between the predicted probabilities and actual incidence rates for the XGBoost model, and decision curve analysis demonstrated that it provided the highest clinical net benefit across most threshold ranges. SHAP analysis revealed that the top three variables contributing the most to the XGBoost model's prediction of postoperative adverse prognosis were the systemic immune-inflammation index (SII), prognostic nutritional index (PNI), and age, in descending order.

**Conclusion:**

The XGBoost model constructed using perioperative data can effectively predict adverse postoperative outcomes in patients with breast cancer undergoing surgery, outperforming traditional models and other machine learning approaches. The preoperative SII level is the most critical predictive factor.

## 1 Introduction

Breast cancer is one of the most common malignant tumors in women. Its incidence and mortality rates rank first and fifth, respectively (1). Surgery is currently the preferred treatment for breast cancer. Surgical removal of breast tumors can prevent tumor progression or metastasis, and adjuvant radiotherapy and chemotherapy after surgery can improve prognosis (2). However, long-term follow-up data have shown that even after standardized surgical treatment, patients still face a high risk of recurrence and metastasis (3, 4). Recurrent tumors are usually associated with higher invasiveness and poorer prognoses. Therefore, early prediction and intervention for adverse outcomes, such as postoperative recurrence and metastasis, are crucial. Many studies have explored the factors influencing adverse postoperative outcomes in breast cancer, including recurrence and metastasis (5, 6). Most of these studies employed traditional methods, including univariate and multivariate analyses. Although these methods help clarify the pathological characteristics of breast cancer, they lack sufficient specificity and sensitivity. As a result, significant challenges remain in predicting individual patients prognosis and creating personalized treatment plans and follow-up schedules.

With the development of data science and computing technology, machine learning techniques have been increasingly applied to the analysis of risk factors and construction of predictive models for adverse postoperative outcomes in various surgical specialties. Studies have shown that machine learning demonstrates a stronger predictive power than traditional methods when processing large volumes of data, particularly in the presence of complex relationships among variables (7). However, the application of machine learning in the medical field is limited. Its “black box” nature—that is, difficulty in understanding the underlying decision-making process—restricts its widespread clinical adoption (8). The Shapley Additive exPlanations (SHAP), based on game theory principles, can quantify the contribution of each feature to the prediction outcome. This approach not only identifies key risk factors through global analysis but also clarifies the rationale for individual predictions via local interpretation, thereby endowing “black box” models with clinical interpretability (9).

Although some studies have applie machine learning to breast cancer prognosis prediction, most models rely on unconventional data, such as genomics (10), which limits their clinical applicability. Other studies focus only on comparing model performance (11) and lack an in-depth interpretation of how decision are made. There remains a lack of research that systematically utilizes readily available perioperative data. Few studies comprehensively compare multiple machine learning algorithms or deeply integrate SHAP interpretability techniques to explain the factors driving predictions. In this study, we integrated several machine learning algorithms, including extreme gradient boosting (XGBoost), random forest (RF), gradient boosting machine (GBM), support vector machine (SVM), and logistic regression (LR), to construct a predictive model for composite postoperative outcomes in breast cancer. Through model comparison and SHAP interpretability analysis, our aim was to accurately identify high-risk patients and clarify key risk factors. This provids an evidence-based foundation for precise clinical prevention and treatment.

## 2 Materials and methods

### 2.1 Clinical data

This was a retrospective, cohort study. A total of 433 patients with breast cancer who underwent initial surgical treatment at the Chongqing University Three Gorges Hospital between May 2020 and May 2024 were enrolled as the modeling set, and 200 patients with breast cancer who underwent the same type of surgery at the First Affiliated Hospital of Changsha Medical University during the same period were included in the external validation set. The study protocol was reviewed and approved by the Ethics Committees of both hospitals. The ethics committee waived the requirement for informed consent for the retrospective analysis of anonymized clinical data.

Inclusion criteria were: (1) female patients with primary breast cancer confirmed by postoperative pathology who underwent initial surgical treatment, including breast-conserving surgery, simple mastectomy, or modified radical mastectomy; (2) patients who underwent traditional open surgery; (3) patients aged 18–80 years; (4) patients with accessible and complete perioperative data, including preoperative clinicopathological data, laboratory test results, surgical treatment details, and postoperative final pathological reports; and (5) patients with clear and complete follow-up information.

Exclusion criteria were: (1) history of other malignant tumors; (2) patients who received neoadjuvant chemotherapy or neoadjuvant targeted therapy before surgery; (3) patients with missing key perioperative data or incomplete follow-up information; (4) patients who only underwent palliative surgery or biopsy; (5) patients with recurrent breast cancer; (6) patients with severe systemic diseases (e.g., cerebral infarction, myocardial infarction, severe heart failure, or severe infectious diseases); (7) refusal to cooperate with treatment or follow-up; (8) patients with known distant metastasis before surgery; (9) patients with severe mental illness or psychological disorders; and (10) patients with bilateral breast cancer.

### 2.2 Follow-up and endpoint events

After breast cancer surgery, all patients were evaluated for adjuvant therapy (chemotherapy, radiotherapy, and/or endocrine therapy) based on multidisciplinary discussions and international guidelines (12). All patients were followed up every 3–6 months for the first 24 months from the date of surgery; from 25 to 60 months, follow-up was conducted every 6–12 months, and after 60 months, the interval was adjusted to every 24 months (13). The follow-up plan included a minimum of local and regional examinations and medical history collection, and serum tumor marker testing and imaging examinations were added when necessary. The follow-up cut-off date was May 2025. The endpoint event was the occurrence of adverse postoperative prognosis.

Adverse postoperative outcomes were defined as composite endpoints, including locoregional recurrence, systemic recurrence, contralateral new primary breast cancer, or breast cancer–related death after surgery. Locoregional recurrence refers to disease recurrence in the breast, chest wall, supraclavicular or infraclavicular regions, or regional lymph nodes, as defined by the TNM staging system of the American Joint Committee on Cancer (AJCC) TNM staging system (14). Systemic recurrence, as defined by M stage, refers to disease recurrence in distant lymph nodes or other organs (14). Recurrence was confirmed by more than two gynecologic oncologists through physical examination, biochemical indicators, imaging examinations, and pathological biopsy. Patients were stratified into adverse and favorable prognosis groups based on the occurrence of postoperative adverse events during follow-up.

### 2.3 Data collection

Based on a literature review and consultation with experts from our hospital, we extracted key perioperative and follow-up data for patients from the hospital's electronic medical record and specialized information systems, which mainly included the following: (1) demographic information: age, body mass index (BMI), menstrual status, family history of breast cancer, smoking and drinking history, and comorbidities (diabetes, hypertension, and hyperlipidemia); (2) preoperative laboratory test indicators: white blood cell count (WBC), platelet count (PLT), serum albumin (ALB), carbohydrate antigen 153 (CA153), carcinoembryonic antigen (CEA), red blood cell distribution width coefficient of variation (RDW-CV), lymphocyte count (LYM), and neutrophil count (NEUT). The systemic immune-inflammation index (SII) and prognostic nutritional index (PNI) were calculated using the following formulas: SII = (PLT × NEUT)/LYM, and PNI = ALB + 5 × LYM; (3) postoperative histopathological examination indicators: affected side, TNM stage, tumor diameter, number of lymph node metastases, lymphovascular invasion (LVI) status, histological type, histological grade, estrogen receptor (ER) status, progesterone receptor (PR) status, human epidermal growth factor receptor 2 (HER2) status, tumor proliferation index (Ki-67) and molecular subtype. Histological types were classified according to the World Health Organization (WHO) classification criteria for breast cancer. Histological grades included well differentiated (grade I), moderately differentiated (grade II), poorly differentiated (grade III), and others. Molecular subtypes were divided into four core types based on the status of ER, PR, HER2, and Ki-67: ① Luminal A: ER and/or PR positive, HER2 negative, and low Ki-67 expression (<14%); ② Luminal B: ER-and/or PR-positive and HER2 positive (regardless of Ki-67 level); b. ER and/or PR positive, HER2 negative, with either high Ki-67 expression (≥14%) or low PR expression; ③ HER2-positive: ER-negative, PR-negative, and HER2 positive; and ④ triple-negative breast cancer (TNBC): ER, PR, and HER2 negative; (4) surgical treatment information: surgical methods, postoperative complications, and details of postoperative adjuvant radiotherapy, chemotherapy, and endocrine therapy; and (5) Follow-up information: date of surgery, date of first diagnosis of recurrence or metastasis, date of death, and date of the last follow-up. Disease-free survival (DFS) was calculated based on follow-up data and defined as the time from the date of surgery to the first occurrence of local recurrence, distant metastasis, or death from any cause. For patients who did not experience an event, survival time was treated as censored data and calculated until the date of the last follow-up or the data cutoff date of this study (May 31, 2025), whichever occurred later.

### 2.4 Model construction and evaluation

During the variable screening stage, univariate analysis was first used to screen all candidate independent variables, with a threshold of *P* < 0.05 for inclusion. This approach was selected based on the following considerations: given the limited sample size, univariate analysis allows for rapid identification of outcome-related variables while controlling model complexity, effectively reducing the risk of overfitting. Additionally, this method yields intuitive results with strong clinical interpretability and is a commonly used strategy for variable screening in clinical prediction modeling.

Data from the modeling set (*n* = 443) were randomly stratified, sampled, and divided into a training set (*n* = 310) and an internal validation set (*n* = 133) at a ratio of 7:3. In the training set, five algorithms, XGBoost, RF, GBM, SVM, and LR, were used to construct predictive models for adverse postoperative outcomes. The hyperparameters were optimized using 5-fold cross-validation and a grid search. The predictive performance of each model was evaluated using an internal validation set, and its generalizability and stability were tested using an external validation set (*n* = 200). The discriminative ability of the models was assessed by calculating the area under the receiver operating characteristic (ROC) curve (AUC), sensitivity, specificity, accuracy, F1 score, and kappa coefficient. Calibration curves were used to evaluate the consistency between the predicted probabilities of the models and actual occurrence probability. Decision curve analysis (DCA) was employed to assess the net benefit of the models across different risk thresholds, thereby quantifying their clinical application value in decision-making. To enhance model interpretability, SHAP was used to rank the global importance of all features, thereby revealing the role of key predictors in a comprehensive context. Additionally, SHAP interaction analysis was performed to explore synergistic effects and potential interactions among variables. In the training set, the optimal cutoff value for the risk score of the best-performing model was determined based on the Youden index maximization principle. Using this cut-off, patients in the external validation set were stratified into high- and low-risk groups. Disease-free survival (DFS) curves were plotted using the Kaplan–Meier method, and survival differences between the high- and low-risk groups were compared using the log-rank test. Furthermore, a Cox proportional hazards regression model was constructed to calculate the hazard ratio (HR) and 95% confidence interval (CI) to quantify risk differences. The concordance index (C-index) was used to evaluate the overall discriminative ability of the model. To address the class imbalance caused by the low incidence of positive events, the SMOTE oversampling technique was applied to generate a balanced dataset, and the performance of models trained on the balanced dataset was compared with that of models trained on the original imbalanced dataset.

### 2.5 Statistical methods

Data processing and analysis were performed using SPSS 23.0 and the R language (version 4.2.1). Normally distributed continuous variables are described as mean ± standard deviation (*x¯* ± *s*), and comparisons between groups were conducted using the independent samples *t*-test or one-way ANOVA. Categorical variables are presented as numbers (*n*) and percentages (%), and group comparisons were performed using the chi-square test or Fisher's exact test. Machine learning classifiers were constructed and implemented using the following R packages: *xgboost*, *randomForest*, *gbm*, *stats*, *caret*, and *e1071*. Statistical significance was defined as a two-sided *p*-value of <0.05.

Based on the one-in-ten rule for the development of clinical prediction models (i.e., 10 event samples are required for each independent variable, EPV = 10) and assuming that the final model includes six key predictive variables, at least 60 event samples are required. Combined with the reported postoperative recurrence rate of approximately 20% for early breast cancer in the literature (15), the total sample size required for model development was calculated to be at least 60/20% = 300 cases. The training set used for modeling in this study included 310 patients, meeting the minimum sample size requirement.

The core objective of external validation is to evaluate the model performance in a new population, and the number of event samples should be comparable to that of the modeling set, or at least half of the modeling set. Given that the modeling set required 60 event samples, the external validation set should include at least 30–60 event samples. Based on a 20% recurrence rate after surgery for early breast cancer, the total sample size required for external validation was at least 30/20% (150 cases). The external validation sample size in this study was 200 cases, which met this minimum requirement.

## 3 Results

### 3.1 Clinical characteristics of patients

A total of 643 patients with breast cancer who underwent surgery were included, of whom 109 (16.95%) experienced adverse postoperative outcomes and 534 (83.05%) had favorable prognoses. There were no statistically significant differences in clinical characteristics or postoperative outcomes among the patients in the training, internal validation, and external validation sets (*P* > 0.05), as shown in Table 1.

**Table 1:** Clinical data characteristics of breast cancer patients in the training set, validation set, and external validation set [*n*(%), *x¯* ± *s*].

Indicators	Training set (*n* = 310)	Validation set (*n* = 133)	External validation set (*n* = 200)	*F*/*χ*^2^ value	*P* value
Age (years)	45.40 ± 6.77	45.23 ± 6.78	45.84 ± 6.72	0.399	0.671
BMI (kg/m^2^)	23.28 ± 2.67	23.27 ± 2.67	23.32 ± 2.41	0.019	0.981
Menstrual status				4.212	0.122
Premenopausal	235 (75.81)	96 (72.18)	135 (67.50)		
Menopause	75 (24.19)	37 (27.82)	65 (32.50)		
Family history of breast cancer				0.193	0.908
No	259 (83.55)	112 (84.21)	170 (85.00)		
Yes	51 (16.45)	21 (15.79)	30 (15.00)		
Diabetes mellitus				0.400	0.819
No	275 (88.71)	116 (87.22)	174 (87.00)		
Yes	35 (11.29)	17 (12.78)	26 (13.00)		
Hypertension				0.744	0.689
No	270 (87.10)	118 (88.72)	171 (85.50)		
Yes	40 (12.90)	15 (11.28)	29 (14.50)		
Hyperlipidemia				3.271	0.195
No	264 (85.16)	114 (85.71)	181 (90.50)		
Yes	46 (14.84)	19 (14.29)	19 (9.50)		
WBC (× 10^9^/L)	14.60 ± 3.67	14.17 ± 4.19	14.56 ± 3.70	0.638	0.529
PLT (× 10^9^/L)	319.28 ± 61.08	321.74 ± 58.11	320.17 ± 63.79	0.075	0.928
ALB (g/L)	30.79 ± 6.32	31.27 ± 6.04	30.01 ± 5.47	1.918	0.148
RDW-CV (%)	13.93 ± 1.56	13.99 ± 1.44	14.10 ± 1.41	0.761	0.468
LYM (× 10^9^/L)	5.48 ± 1.48	5.40 ± 1.55	5.71 ± 1.26	2.385	0.093
NEUT (× 10^9^/L)	9.29 ± 2.75	9.02 ± 2.87	9.68 ± 2.48	2.533	0.080
SII	528.00 ± 39.66	522.73 ± 31.34	528.16 ± 34.70	1.124	0.326
PNI	58.17 ± 5.01	58.27 ± 5.74	58.57 ± 5.28	0.360	0.698
CA153 (U/mL)	53.34 ± 8.78	54.35 ± 8.87	53.87 ± 9.18	0.638	0.529
CEA (ng/mL)	7.22 ± 1.23	7.33 ± 1.09	7.26 ± 1.14	0.407	0.666
Affected side				0.465	0.793
Left side	158 (50.97)	65 (48.87)	96 (48.00)		
Right side	152 (49.03)	68 (51.13)	104 (52.00)		
TNM staging				5.064	0.281
StageⅠ	82 (26.45)	42 (31.58)	46 (23.00)		
StageⅡ	160 (51.61)	65 (48.87)	118 (59.00)		
StageⅢ	68 (21.94)	26 (19.55)	36 (18.00)		
Tumor diameter				0.144	0.930
<4 cm	233 (75.16)	98 (73.68)	148 (74.00)		
≥4 cm	77 (24.84)	35 (26.32)	52 (26.00)		
LVI positivity				1.839	0.399
No	273 (88.06)	121 (90.98)	183 (91.50)		
Yes	37 (11.94)	12 (9.02)	17 (8.50)		
ER-positive				1.362	0.506
No	74 (23.87)	26 (19.55)	41 (20.50)		
Yes	236 (76.13)	107 (80.45)	159 (79.50)		
PR-positive				0.366	0.833
No	116 (37.42)	47 (35.34)	70 (35.00)		
Yes	194 (62.58)	86 (64.66)	130 (65.00)		
HER2-positive				1.821	0.402
No	260 (83.87)	118 (88.72)	172 (86.00)		
Yes	50 (16.13)	15 (11.28)	28 (14.00)		
Ki-67-positive				0.232	0.891
No	237 (76.45)	104 (78.20)	152 (76.00)		
Yes	73 (23.55)	29 (21.80)	48 (24.00)		
Molecular subtype				2.543	0.864
Luminal A subtype	155 (50.00)	75 (56.39)	106 (53.00)		
Luminal B subtype	81 (26.13)	32 (24.06)	53 (26.50)		
HER2-positive subtype	25 (8.06)	7 (5.26)	12 (6.00)		
TNBC	49 (15.81)	19 (14.29)	29 (14.50)		
Surgical method				5.655	0.226
Partial mastectomy	98 (31.61)	48 (36.09)	74 (37.00)		
Total mastectomy	130 (41.94)	61 (45.86)	87 (43.50)		
Modified radical mastectomy	82 (26.45)	24 (18.05)	39 (19.50)		
Number of lymph node metastases				2.120	0.346
<4 pieces	270 (87.10)	112 (84.21)	165 (82.50)		
≥4 pieces	40 (12.90)	21 (15.79)	35 (17.50)		
Histological type				2.553	0.863
Invasive ductal carcinoma	189 (60.97)	79 (59.40)	126 (63.00)		
Carcinoma *in situ*	66 (21.29)	33 (24.81)	37 (18.50)		
Invasive lobular carcinoma	21 (6.77)	10 (7.52)	15 (7.50)		
Malignant phyllodes tumor	34 (10.97)	11 (8.27)	22 (11.00)		
Histological grades				1.204	0.877
Grade I	85 (27.42)	41 (30.83)	60 (30.00)		
Grade II	108 (34.84)	48 (36.09)	67 (33.50)		
Grade III	117 (37.74)	44 (33.08)	73 (36.50)		
Adjuvant radiotherapy				0.561	0.755
No	45 (14.52)	16 (12.03)	26 (13.00)		
Yes	265 (85.48)	117 (87.97)	174 (87.00)		
Adjuvant chemotherapy				0.529	0.768
No	37 (11.94)	16 (12.03)	20 (10.00)		
Yes	273 (88.06)	117 (87.97)	180 (90.00)		
Adjuvant endocrine therapy				1.362	0.506
No	74 (23.87)	26 (19.55)	41 (20.50)		
Yes	236 (76.13)	107 (80.45)	159 (79.50)		
Postoperative complications				0.230	0.892
No	246 (79.35)	108 (81.20)	161 (80.50)		
Yes	64 (20.65)	25 (18.80)	39 (19.50)		
Postoperative prognosis				0.282	0.868
Favorable	255 (82.26)	111 (83.46)	168 (84.00)		
Adverse	55 (17.74)	22 (16.54)	32(16.00)		

BMI, body mass index; WBC, white blood cell; PLT, platelet count; ALB, serum albumin; RDW-CV, red blood cell distribution width coefficient of variation; LYM, lymphocyte count; NEUT, neutrophil count; SII, systemic immune-inflammation index; PNI, prognostic nutritional index. CA153, carbohydrate antigen 153; CEA, carcinoembryonic antigen; TNM, tumor-node-metastasis staging; LVI, Lymphovascular invasion; ER, estrogen receptor; PR, progesterone receptor; HER2, human epidermal growth factor receptor 2; Ki-67, tumor proliferation index; TNBC, triple-negative breast cancer.

### 3.2 Univariate analysis of adverse postoperative outcomes in breast cancer

In the training set, 255 patients (82.26%) with favorable postoperative outcomes were included in the favorable prognosis group and 55 patients (17.74%) with adverse postoperative outcomes were included in the adverse prognosis group. Compared with the favorable prognosis group, the adverse prognosis group had significantly higher age, a higher proportion of TNM stage III, tumor diameter ≥4 cm, lymphovascular invasion (LVI), HER2 positivity, lymph node metastasis ≥4, higher SII levels (all *P* < 0.05), and significantly lower PNI levels (*P* < 0.05). The detailed results are presented in Table 2.

**Table 2:** Results of univariate analysis on adverse prognosis [*n* (%), *x¯* ± *s*].

Indicators	Favorable prognosis group (*n* = 255)	Adverse prognosis group (*n* = 55)	*t*/χ^2^ value	*P* value
Age (years)	45.82 ± 6.67	43.44 ± 6.94	2.385	0.018
BMI (kg/m^2^)	23.27 ± 2.51	23.35 ± 2.64	0.216	0.829
Menstrual status			0.641	0.423
Premenopausal	191 (74.90)	44 (80.00)		
Menopause	64 (25.10)	11 (20.00)		
Family history of breast cancer			0.146	0.703
No	214 (83.92)	45 (81.82)		
Yes	41 (16.08)	10 (18.18)		
Diabetes mellitus			0.707	0.400
No	228 (89.41)	47 (85.45)		
Yes	27 (10.59)	8 (14.55)		
Hypertension			0.712	0.399
No	224 (87.84)	46 (83.64)		
Yes	31 (12.16)	9 (16.36)		
Hyperlipidemia			0.591	0.442
No	219 (85.88)	45 (81.82)		
Yes	36 (14.12)	10 (18.18)		
WBC (× 10^9^/L)	14.44 ± 3.60	15.32 ± 3.96	1.608	0.109
PLT (× 10^9^/L)	319.73 ± 62.70	317.16 ± 53.37	0.283	0.778
ALB (g/L)	31.06 ± 6.56	29.56 ± 4.92	1.919	0.058
RDW-CV (%)	13.87 ± 11.53	14.23 ± 1.67	1.590	0.113
LYM (× 10^9^/L)	5.51 ± 1.54	5.33 ± 1.16	0.981	0.329
NEUT (× 10^9^/L)	9.28 ± 2.80	9.33 ± 2.53	0.119	0.905
SII	524.60 ± 1.85	543.73 ± 62.63	2.204	0.031
PNI	58.59 ± 4.95	56.19 ± 4.87	3.268	0.001
CA153 (U/mL)	53.05 ± 9.01	54.69 ± 7.53	1.261	0.208
CEA (ng/mL)	7.15 ± 1.18	7.52 ± 1.38	1.809	0.075
Affected side			1.392	0.238
Left side	126 (49.41)	32 (58.18)		
Right side	129 (50.59)	23 (41.82)		
TNM staging			14.075	0.001
Stage I	77 (30.20)	5 (9.09)		
Stage II	130 (50.98)	30 (54.55)		
Stage III	48 (18.82)	20 (36.36)		
Tumor diameter			12.655	<0.001
<4 cm	202 (79.22)	31 (56.36)		
≥4 cm	53 (20.78)	24 (43.64)		
LVI positivity			11.626	0.001
No	232 (90.98)	41 (74.55)		
Yes	23 (9.02)	14 (25.45)		
ER-positive			2.886	0.089
No	56 (21.96)	18 (32.73)		
Yes	199 (78.04)	37 (67.27)		
PR-positive			2.772	0.096
No	90 (35.29)	29 (52.73)		
Yes	165 (64.71)	26 (47.27)		
HER2-positive			4.298	0.038
No	219 (85.88)	41 (74.55)		
Yes	36 (14.12)	14 (25.45)		
Ki-67-positive			0.550	0.458
No	197 (77.25)	45 (81.82)		
Yes	58 (22.75)	10 (18.18)		
Molecular subtype			5.617	0.132
Luminal A subtype	128 (50.20)	27 (49.09)		
Luminal B subtype	71 (27.84)	10 (18.18)		
HER2-positive subtype	21 (8.24)	4 (7.27)		
TNBC	35 (13.73)	14 (25.45)		
Surgical method			0.311	0.856
Partial mastectomy	82 (32.16)	16 (29.09)		
Total mastectomy	107 (41.96)	23 (41.82)		
Modified radical mastectomy	66 (25.88)	16 (29.09)		
Number of lymph node metastases			6.854	0.009
<4 pieces	228 (89.41)	42 (76.36)		
≥4 pieces	27 (10.59)	13 (23.64)		
Histological type,			6.927	0.074
Invasive ductal carcinoma	147 (57.65)	42 (76.36)		
Carcinoma *in situ*	59 (23.14)	7 (12.73)		
Invasive lobular carcinoma	18 (7.06)	3 (5.45)		
Malignant phyllodes tumor	31 (12.16)	3 (5.45)		
Histological grades			4.448	0.108
Grade I	75 (29.41)	10 (18.18)		
Grade II	90 (35.29)	18 (32.73)		
Grade III	90 (35.29)	27 (49.09)		
Adjuvant radiotherapy			0.701	0.402
No	39 (15.29)	6 (10.91)		
Yes	216 (84.71)	49 (89.09)		
Adjuvant chemotherapy			1.383	0.240
No	33 (12.94)	4 (7.27)		
Yes	222 (87.06)	51 (92.73)		
Adjuvant endocrine therapy			2.886	0.089
No	56 (21.96)	18 (32.73)		
Yes	199 (78.04)	37 (67.27)		
Postoperative complications			0.365	0.546
No	204 (80.00)	42 (76.36)		
Yes	51 (20.00)	13(23.64)		

BMI, body mass index; WBC, white blood cell; PLT, platelet count; ALB, serum albumin; RDW-CV, red blood cell distribution width coefficient of variation; LYM, lymphocyte count; NEUT, neutrophil count; SII, systemic immune-inflammation index; PNI, prognostic nutritional index. CA153, carbohydrate antigen 153; CEA, carcinoembryonic antigen; TNM, tumor-node-metastasis staging; LVI, Lymphovascular invasion; ER, estrogen receptor; PR, progesterone receptor; HER2, human epidermal growth factor receptor 2; Ki-67, tumor proliferation index; TNBC, triple-negative breast cancer.

### 3.3 Logistic regression analysis of adverse postoperative outcomes in breast cancer

Given that the TNM staging system integrates information from its constituent components (tumor diameter and number of lymph node metastases), and to avoid multicollinearity, we selected this composite indicator, rather than its individual components, to represent anatomical tumor burden. Multicollinearity diagnostics showed that the variance inflation factor (VIF) values for age, TNM stage, LVI, HER2 status, SII, and PNI ranged from 1.012 to 1.033, and tolerance values ranged from 0.968 to 0.988, all within acceptable thresholds (VIF < 5, tolerance > 0.1), indicating no severe multicollinearity among these factors.

Using postoperative prognosis (favorable = 0, adverse = 1) as the dependent variable and age (continuous variable), TNM stage (stage I = 0, stage II = 1, stage III = 2), LVI positivity (no = 0, yes = 1), HER2 positivity (no = 0, yes = 1), SII (continuous variable), and PNI (continuous variable) as independent variables, a multivariate logistic regression analysis was performed. The results showed that TNM stages II and III (compared with stage I), LVI positivity, HER2 positivity, and high SII were independent risk factors for adverse postoperative prognosis (*P* < 0.05), whereas older age and high PNI were protective factors. The detailed results are presented in Table 3.

**Table 3:** Results of multivariate logistic regression analysis on adverse postoperative outcomes.

Independent variable	*B*	*S.E*	*Wald χ* ^2^	*P*	*OR*	95%CI	
						Lower limit	Upper limit
Age (years)	−0.059	0.025	5.317	0.021	0.943	0.897	0.991
TNM (stage II)	1.435	0.540	7.064	0.008	4.198	1.458	12.094
TNM (stage III)	1.830	0.570	10.309	0.001	6.233	2.040	19.046
LVI positivity	1.208	0.433	7.784	0.005	3.347	1.432	7.821
HER2 positivity	0.839	0.399	4.432	0.035	2.314	1.060	5.054
SII	0.013	0.005	7.797	0.005	1.013	1.004	1.022
PNI	−0.092	0.033	7.672	0.006	0.912	0.854	0.973

95% CI, 95% confidence interval; VIF, variance inflation factor; TNM, tumor-node-metastasis staging; LVI, lymphovascular invasion; HER2, human epidermal growth factor receptor 2; SII, systemic immune inflammation index; PNI, prognostic nutritional index.

### 3.4 Model construction and evaluation

Based on the significant variables identified by the univariate analysis, this study constructed five models (LR, XGBoost, RF, SVM, and GBM) to predict adverse postoperative outcomes in patients undergoing breast cancer surgery.

ROC curve analysis showed that in the training set, the AUC values of the models ranged from 0.766 to 0.992, with RF achieving the highest AUC (0.992) and LR the lowest (0.766). In the internal validation set, the AUC values ranged from 0.745 to 0.840, with XGBoost achieving the highest AUC (0.840) and SVM the lowest AUC (0.745). In the external validation set, the AUC values ranged from 0.745 to 0.780, with XGBoost achieving the highest AUC (0.780) and LR the lowest AUC (0.745). A comprehensive comparison of model performance revealed that XGBoost achieved the highest AUC in both the internal and external validation sets, demonstrated the most stable performance across the three datasets, and attained the highest specificity (0.881) and F1 score (0.514) in the external validation set, which is of significant clinical importance for identifying patients with postoperative adverse prognosis. The detailed results are presented in Table 4.

**Table 4:** Evaluation of the predictive performance of models on the training set, validation set, and external validation set.

Models	AUC (95%CI)	Accuracy	Sensitivity	Specificity	F1-Measure	Kappa coefficient
Training set						
LR	0.766 (0.696–0.836)	0.842	0.545	0.875	0.329	0.264
XG Boost	0.903 (0.864–0.943)	0.868	0.909	0.773	0.612	0.495
RF	0.992 (0.986–0.998)	0.910	1.000	0.937	0.675	0.631
SVM	0.877 (0.826–0.928)	0.903	0.673	0.953	0.711	0.654
GBM	0.861 (0.814–0.909)	0.852	0.836	0.776	0.583	0.300
Internal validation set						
LR	0.750 (0.614–0.887)	0.887	0.591	0.883	0.545	0.489
XG Boost	0.840 (0.752–0.927)	0.880	0.818	0.730	0.508	0.373
RF	0.826 (0.730–0.922)	0.872	0.682	0.838	0.452	0.393
SVM	0.745 (0.621–0.868)	0.707	0.727	0.703	0.451	0.288
GBM	0.807 (0.708–0.906)	0.850	0.808	0.676	0.550	0.143
External validation set						
LR	0.745 (0.639–0.850)	0.865	0.562	0.869	0.372	0.316
XG Boost	0.780 (0.690–0.870)	0.855	0.656	0.881	0.514	0.292
RF	0.772 (0.683–0.860)	0.860	0.719	0.732	0.364	0.303
SVM	0.754 (0.658–0.849)	0.705	0.688	0.708	0.427	0.265
GBM	0.751 (0.655–0.846)	0.845	0.719	0.732	0.500	0.089

AUC, area under the ROC curve; 95%CI, 95% confidence interval; XGBoost, extreme gradient boosting; RF, random forest; GBM, gradient boosting machine; SVM, support vector machine; LR, logistic regression.

**Figure 1:**
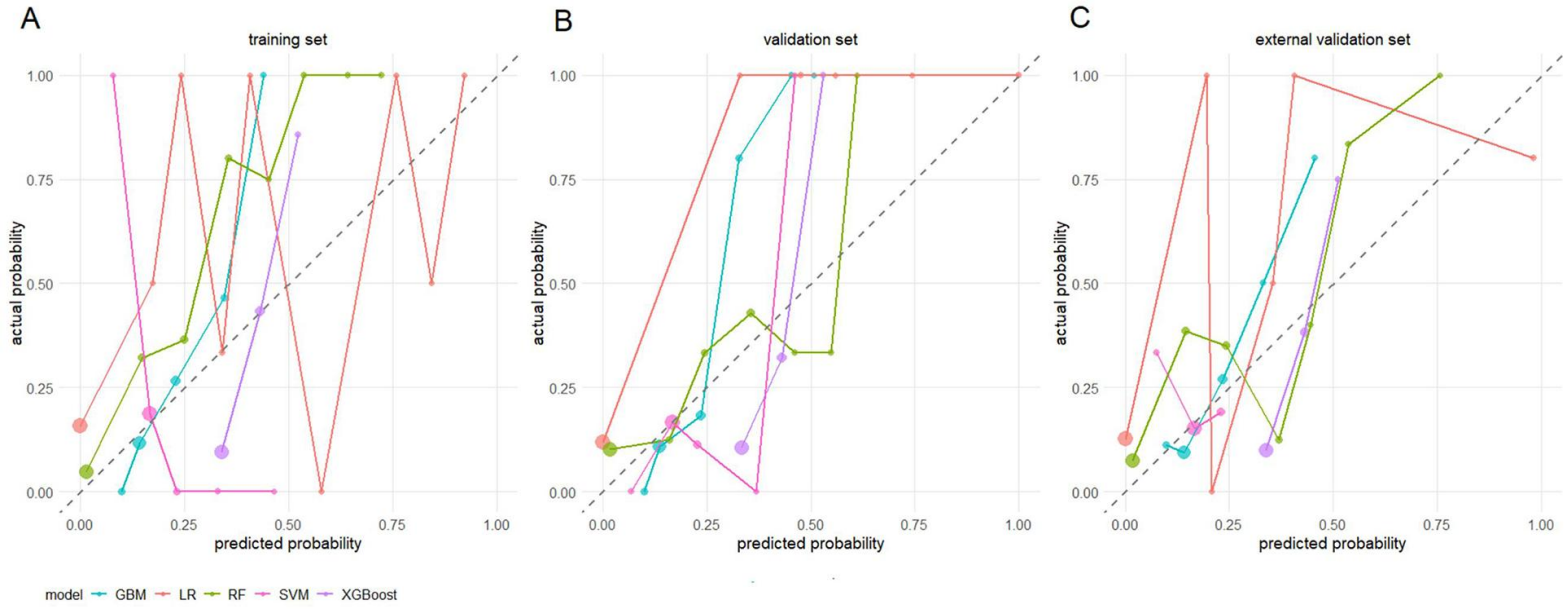
Calibration curves.

**Figure 2:**
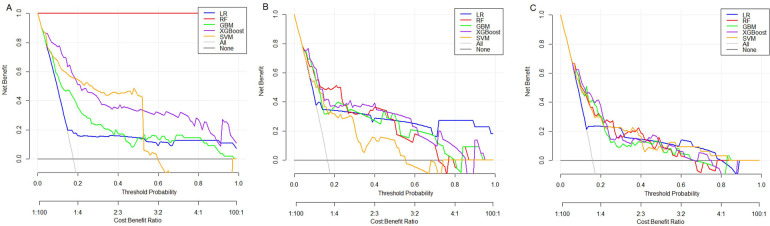
Decision curves [**(A)** training set; **(B)** internal validation set; **(C)** external validation set].

The predictive performance of the models was systematically evaluated using calibration curves and DCA. The calibration curves (Figure 1) showed that compared with the other models, the calibration curves of the XGBoost model in the training, internal validation, and external validation sets were closest to the ideal reference line, indicating the best agreement between the predicted and observed probabilities. DCA demonstrated that the XGBoost model achieved the highest net clinical benefit across most threshold ranges in the training, internal validation, and external validation sets (Figure 2). The XGBoost model exhibited the best overall predictive performance considering discrimination, calibration, and clinical applicability.

On the SMOTE-balanced dataset, the XGBoost model demonstrated superior predictive performance in both the internal and external validation sets (AUC: 0.802 for the internal validation set and 0.778 for the external validation set), outperforming the other comparative models. The performance of the XGBoost model trained on the SMOTE-balanced dataset showed no statistically significant differences compared with the original model in either the internal validation set (AUC: 0.802 vs. 0.840) or external validation set (AUC: 0.778 vs. 0.780) (DeLong test, *p* > 0.05). However, after training on the balanced dataset, the sensitivity of the model in the external validation set increased from 0.656 to 0.781, whereas its specificity decreased from 0.881 to 0.696. Detailed results are presented in Table 5. Overall, XGBoost consistently outperformed other models across both original and SMOTE-balanced datasets and was therefore selected as the optimal model for predicting adverse postoperative outcomes in breast cancer.

**Table 5:** Evaluation of model predictive performance on the SMOTE-balanced dataset.

Models	AUC (95%CI)	Accuracy	Sensitivity	Specificity	F1-Measure	Kappa coefficient
Training set						
LR	0.772 (0.730–0.813)	0.667	0.614	0.714	0.631	0.329
XG Boost	0.994 (0.989–0.998)	0.968	0.955	0.980	0.966	0.936
RF	1.000 (1.000–1.000)	0.998	0.995	1.000	0.998	0.996
SVM	0.889 (0.861–0.917)	0.779	0.705	0.875	0.763	0.556
GBM	0.959 (0.945–0.974)	0.884	0.827	0.933	0.869	0.766
Internal validation set						
LR	0.771 (0.645–0.898)	0.774	0.636	0.802	0.483	0.349
XG Boost	0.802 (0.704–0.901)	0.805	0.727	0.748	0.480	0.362
RF	0.797 (0.679–0.916)	0.857	0.682	0.919	0.558	0.473
SVM	0.751 (0.619–0.882)	0.812	0.636	0.838	0.510	0.396
GBM	0.793 (0.672–0.914)	0.850	0.727	0.775	0.545	0.455
External validation set						
LR	0.744 (0.642–0.846)	0.745	0.562	0.780	0.414	0.265
XG Boost	0.778 (0.688–0.869)	0.805	0.781	0.696	0.480	0.363
RF	0.753 (0.662–0.844)	0.840	0.688	0.720	0.429	0.338
SVM	0.739 (0.643–0.834)	0.765	0.625	0.780	0.447	0.309
GBM	0.771 (0.684–0.859)	0.805	0.906	0.536	0.418	0.301

AUC, area under the ROC curve; 95%CI, 95% confidence interval; XGBoost, extreme gradient boosting; RF, random forest; GBM, gradient boosting machine; SVM, support vector machine; LR, logistic regression.

### 3.5 SHAP interpretation analysis of the model

**Figure 3:**
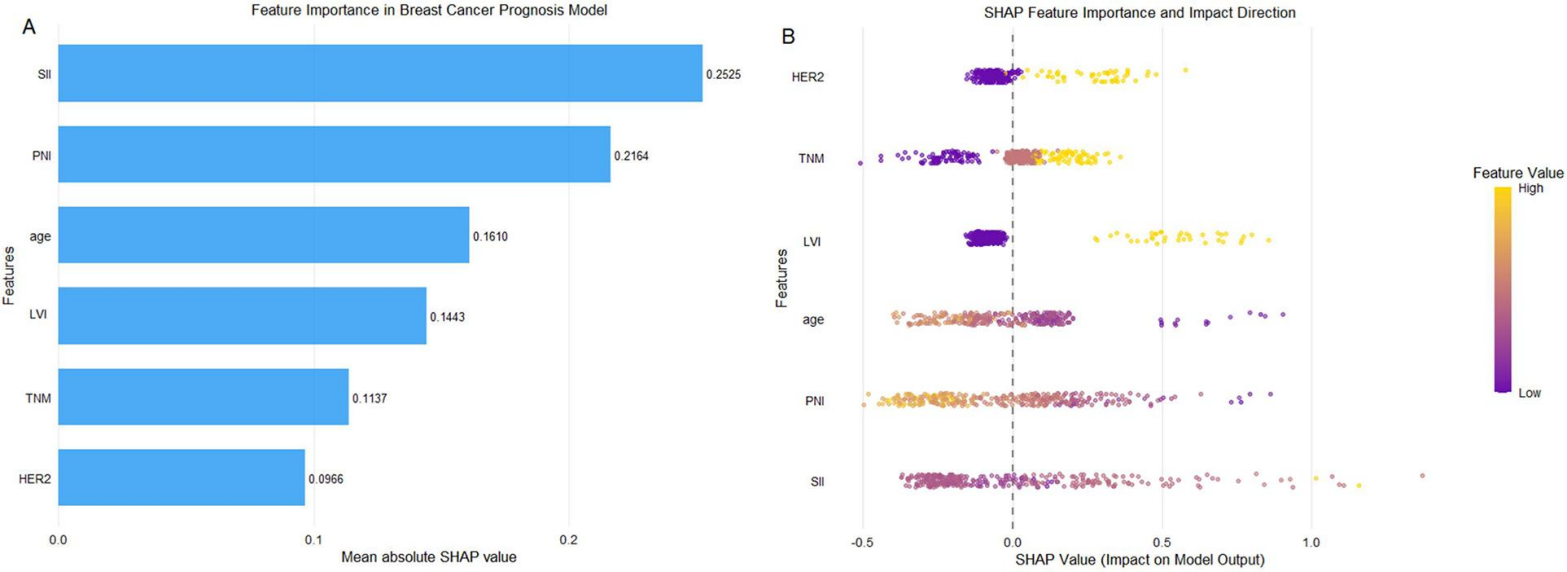
SHAP feature importance of the XGBoost model **(A)** SHAP feature importance bar plot; features are ranked by mean absolute SHAP values from top (most important) to bottom (less important). **(B)** SHAP feature importance bee swarm plot; each point represents the SHAP value for an individual patient, with the color scale indicating the feature value (yellow: high; purple: low). Points to the right of the vertical zero line (SHAP value >0) contribute to higher predicted risk of adverse postoperative outcomes, while points to the left (SHAP value <0) contribute to lower predicted risk. LVI, lymphovascular invasion; TNM, Tumor-Node-Metastasis; HER2, human epidermal growth factor receptor 2; SII, Systemic Immune-Inflammation Index; PNI, Prognostic Nutritional Index.

The XG Boost model was identified as the model with the best predictive performance in this study. To further explain the contribution of each feature to the model's predictions, this study conducted a SHAP analysis; the results are visualized in Figure 3. As shown in the Figure 3A, SII level contributes most significantly to the model's predictions, substantially driving the model's decision-making process. PNI level ranks second in terms of influence, while the average absolute SHAP values for age, LVI, TNM staging, and HER2 positivity decrease in that order, with their importance also decreasing accordingly.

The XG Boost model was identified as the model with the best predictive performance in this study. To further explain the contribution of each feature to the model's predictions, this study conducted a SHAP analysis; the results are visualized in Figure 3. As shown in the SHAP feature importance bar chart (Figure 3A), the SII level exhibited the greatest predictive contribution to the model and played a dominant role in the decision-making process. The PNI was the second most influential feature. The mean absolute SHAP values and the importance of age, LVI, TNM stage, and HER2 positivity showed a sequential decline. As illustrated by the SHAP feature importance Beeswarm plot (Figure 3B), higher SII levels, lower PNI levels, younger age, LVI positivity, higher TNM stages, and HER2 positivity were all associated with an increased risk of adverse postoperative prognosis.

**Figure 4:**
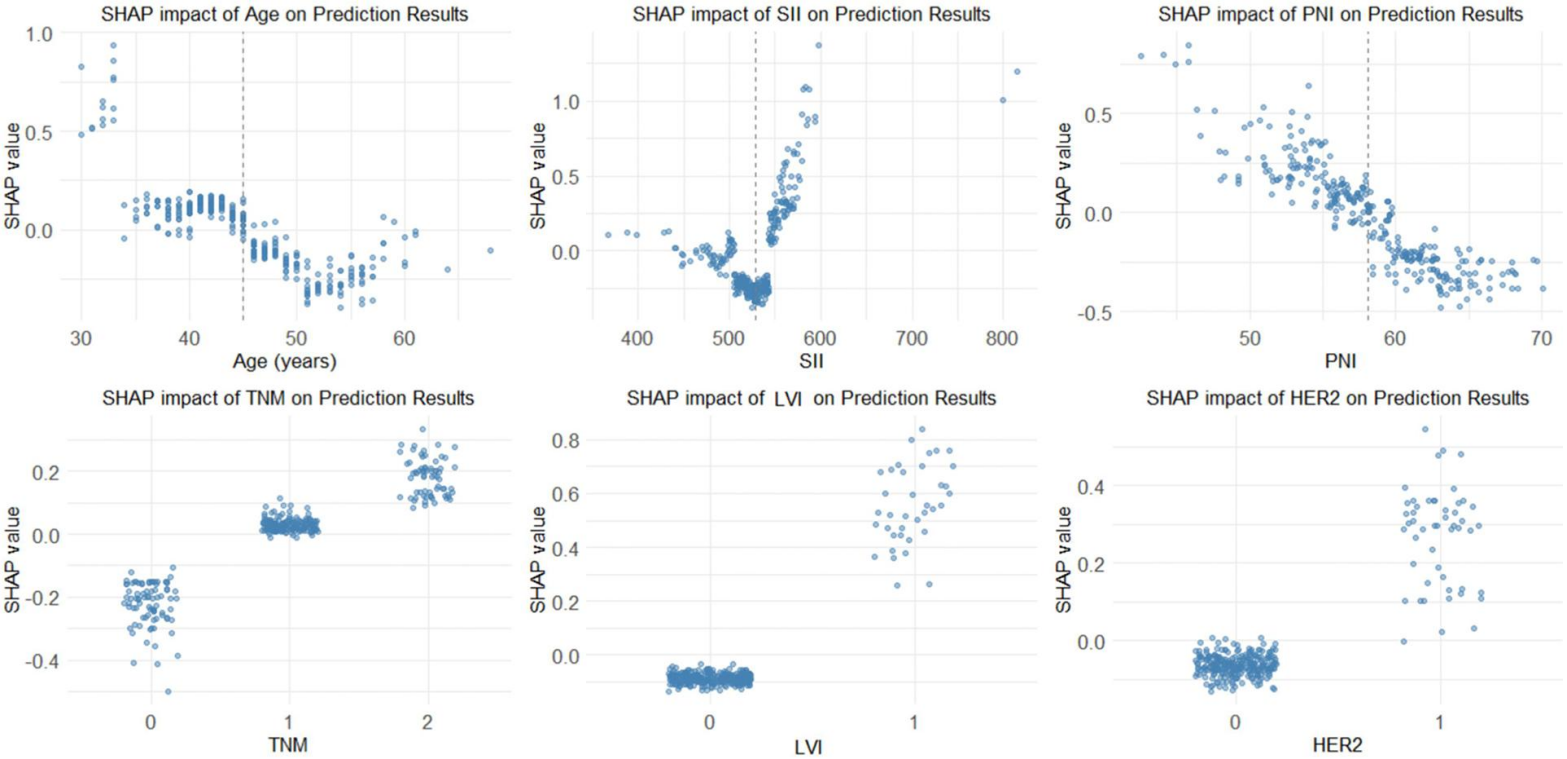
Dependency plots of individual variables. LVI, Lymphovascular Invasion; negative = 0, positive = 1. TNM, Tumor-Node-Metastasis stage; stage Ⅰ = 0, stage Ⅱ = 1, stage Ⅲ = 2. HER2, Human Epidermal Growth Factor Receptor 2; negative = 0, positive = 1. SII, Systemic Immune-Inflammation Index. PNI, Prognostic Nutritional Index.

Based on the SHAP analysis, the effects of different clinical characteristics on the risk of adverse postoperative prognosis in breast cancer can be summarized as follows: SII, PNI, TNM stage, LVI, and HER2 status showed clear linear correlations with the prognostic risk. The SII was positively correlated with risk, with a marked increase observed when the SII was >500. The PNI was negatively correlated with risk, with higher values corresponding to lower risk. More advanced TNM stages, positive LVI status, and positive HER2 status significantly increased the risk of adverse postoperative prognosis. In contrast, age exhibited a stage-specific nonlinear pattern, with the 30–40 and >60 years groups representing high-risk stages, whereas the 40–55 years group showed a relatively lower risk. Detailed results are presented in Figure 4.

**Figure 5:**
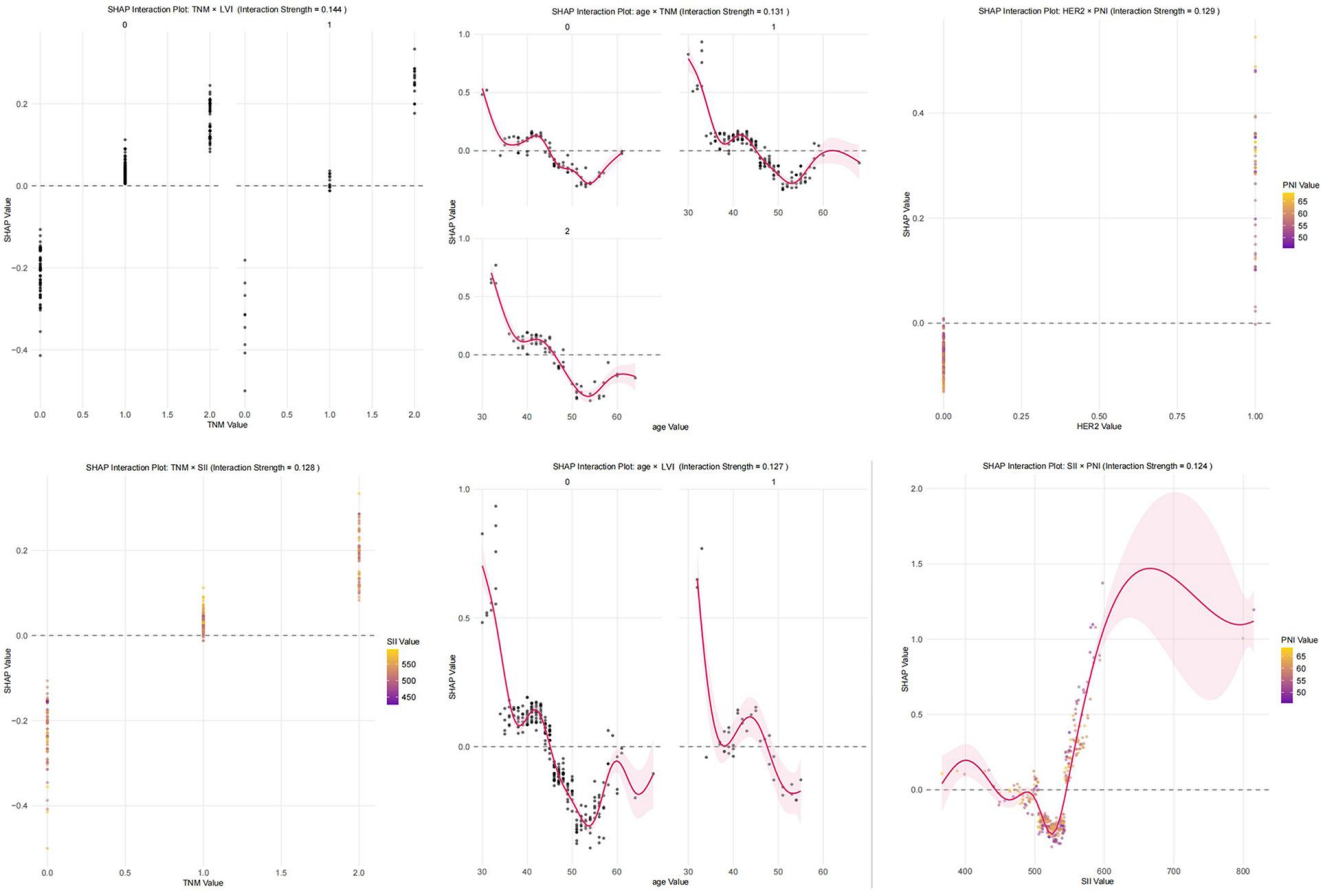
SHAP interaction plots. LVI, Lymphovascular Invasion; negative = 0, positive = 1. TNM, Tumor-Node-Metastasis stage; stage Ⅰ = 0, stage Ⅱ = 1, stage Ⅲ = 2. HER2, Human Epidermal Growth Factor Receptor 2; negative = 0, positive = 1. SII, Systemic Immune-Inflammation Index. PNI, Prognostic Nutritional Index.

In the SHAP interaction analysis, synergistic enhancement effects were generally observed for age, TNM stage, LVI, HER2 status, SII, and PNI in pairwise comparisons, although the overall interaction strength was relatively weak, ranging from 0.011 to 0.144. Among these interactions, the combination of TNM stage and LVI status showed the strongest interaction effect (0.144), indicating that, in cases of advanced tumors combined with LVI, the risk of poor prognosis is synergistically intensified rather than merely additive. The interactions between age and TNM stage, as well as between HER2 status and PNI, were of secondary strength, with interaction intensities of 0.131 and 0.129, respectively. These findings suggest that elderly patients with advanced TNM stages face compounded risks, and patients with HER2-positive status combined with poor nutritional status (low PNI) are at an increased risk of adverse outcomes. Detailed results are shown in Figure 5.

**Figure 6:**
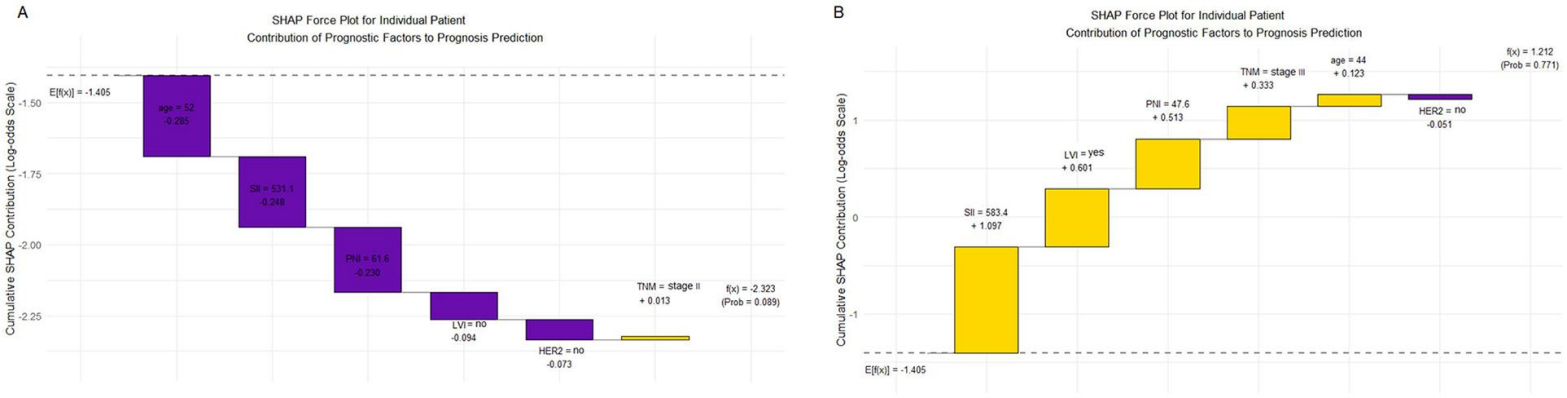
Individual case interpretation of the XGBoost model prediction results **(A)** Patients with favorable postoperative prognosis. **(B)** Patients with adverse postoperative prognosis. LVI, Lymphovascular Invasion; TNM, Tumor-Node-Metastasis stage; HER2, Human Epidermal Growth Factor Receptor 2; SII, Systemic Immune-Inflammation Index. PNI, Prognostic Nutritional Index.

**Figure 7:**
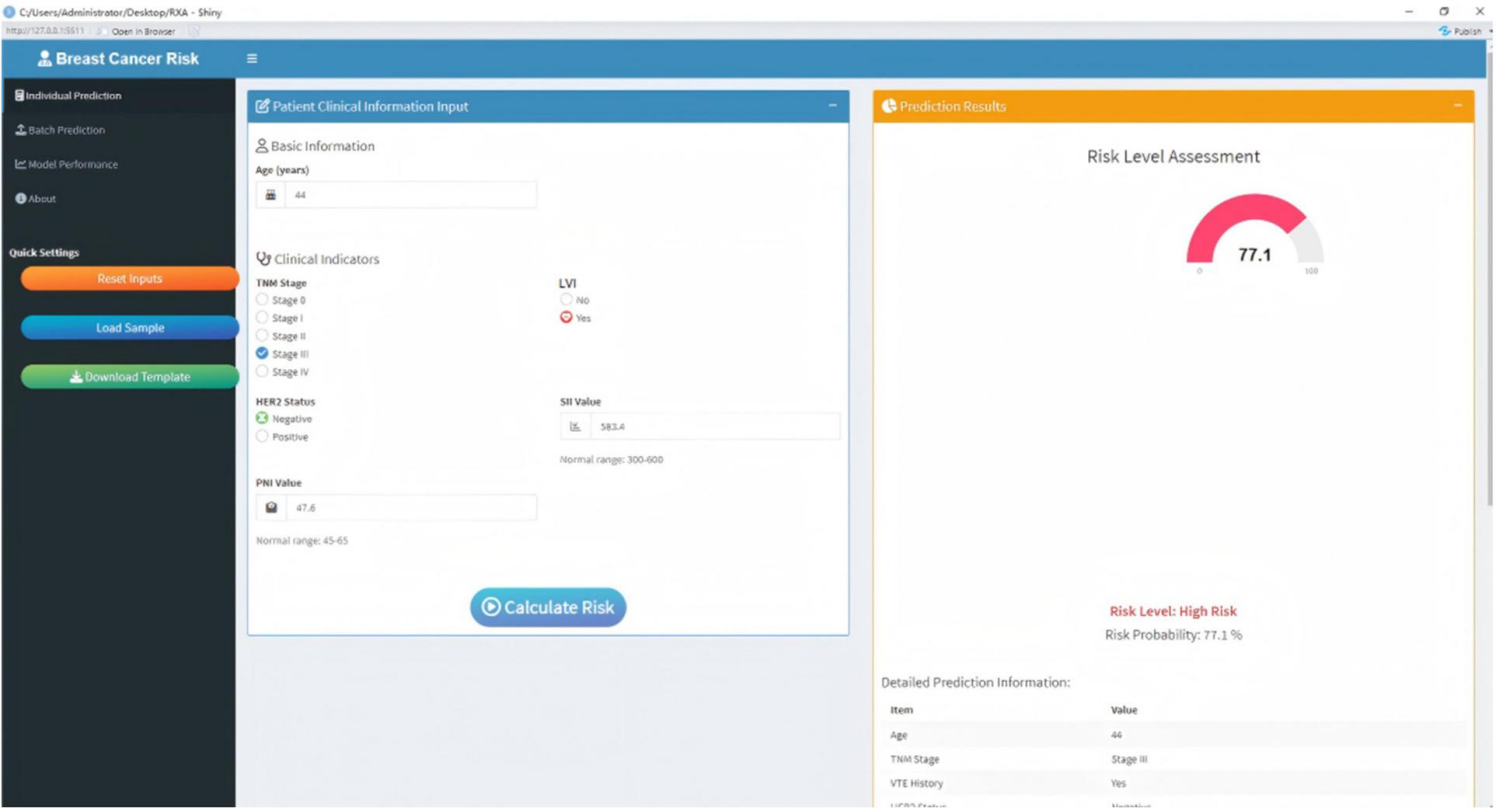
Interactive web-based prediction tool (the sample patient data corresponds to patient B in Figure 6).

Two representative clinical cases were selected for individual prediction and interpretability analyses using the XGBoost model. For Patient A (Figure 6A), with a favorable postoperative prognosis, the SHAP value was negative (−2.323), and the predicted probability was low (0.089). In contrast, Patient B (Figure 6B), with an adverse postoperative prognosis, exhibited a positive SHAP value (1.212) and a markedly higher predicted probability (0.771). The model predictions were consistent with actual clinical prognostic outcomes, supporting the accuracy and interpretability of the model at the individual patient level. To facilitate translation of these findings into clinical practice, an interactive web-based prediction tool was developed using the R Shiny framework (Figure 7). This tool allows clinicians to input patients' clinical characteristics and obtains real-time individualized postoperative risk predictions, enabling its convenient application in multiple clinical settings, including outpatient clinics and inpatient wards. The preliminary deployment access address is http://127.0.0.1:5511.

### 3.6 Risk stratification and survival analysis based on the XGBoost model

**Figure 8:**
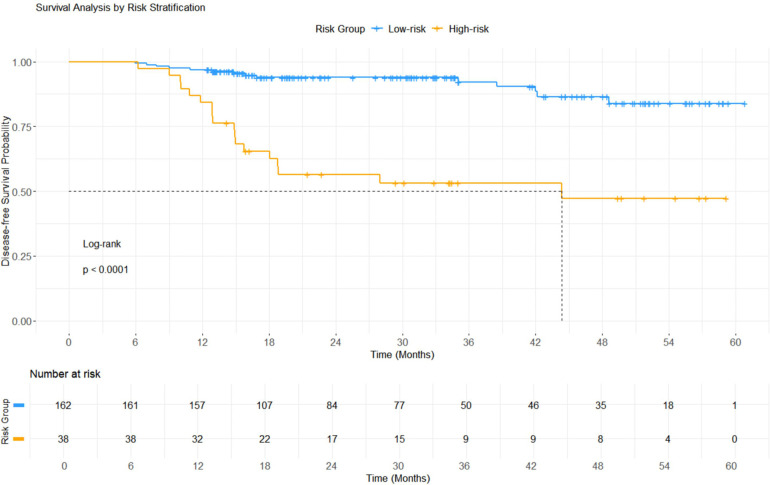
Results of Kaplan-Meier survival curve analysis.

In the external validation cohort (*n* = 200), the risk stratification system based on the XGBoost model, with an optimal cutoff value of 0.266, divided patients into high-risk (*n* = 38) and low-risk (*n* = 162) groups. The median follow-up duration of patients in the external validation cohort was 26.6 months (range, 5.9–60.8 months). The incidence of adverse prognostic events was 47.37% (18/38) in the high-risk group, which was markedly higher than in the low-risk group [8.64% (14/162)]. Survival analysis demonstrated a significant difference in DFS curves between the two groups (log-rank test: *χ*^2^ = 33.7, *p* < 0.0001). The median DFS in the high-risk group was 44.3 months, whereas the median DFS in the low-risk group was not reached. Detailed results are shown in Figure 8.

## 4 Discussion

Currently, machine learning techniques are being increasingly applied to the prediction of breast cancer recurrence and metastasis. Unlike previous studies focusing on medical imaging and pathological images (15, 16–17), this study used easily accessible perioperative clinical and laboratory indicators, with postoperative recurrence and death as the endpoint. We constructed and compared five machine learning prediction models, and adopted SHAP for model interpretation. The results showed that the XGBoost model achieved the best performance, with SHAP analysis identifying SII, PNI, and age as the top contributors to prediction.

Traditional LR analysis identified TNM stage, LVI, HER2 positivity, age, SII, and PNI as independent. predictors of adverse postoperative outcomes. Among these variables, advanced TNM stage, positive LVI, positive HER2 status, and elevated SII level were risk factors, whereas older age and higher PNI level were protective factors, consistent with previous reports (18–21). The consistency of the results across studies reinforces the importance of these prognostic factors in different cohorts. While the LR model performed well in all datasets, the XGBoost, RF, and GBM models showed higher AUC values across all sets, and the SVM model also outperformed LR in the training and external validation sets. Notably, no significant difference in AUC was observed between the RF and XGBoost models across all datasets, consistent with previous studies (22). In the external validation set, the XGBoost model exhibited higher specificity and F1 scores, along with favorable calibration characteristics and net clinical benefit. Considering discrimination, calibration, and clinical utility collectively, the XGBoost model exhibited the best overall predictive performance, consistent with prior studies (23, 24). XGBoost achieved optimal performance mainly due to its built-in regularization, which effectively reduces overfitting and improves model robustness. It can automatically capture complex nonlinear relationships and interactions among clinical features. Furthermore, its efficient computation and built-in cross-validation help balance prediction accuracy and generalization ability with limited and imbalanced clinical data (22, 25). To address class imbalance, we performed a supplementary analysis using SMOTE. The SMOTE-balanced XGBoost model maintained superior performance (internal AUC: 0.802; external AUC: 0.778), with sensitivity improving from 0.656 to 0.781 and specificity decreasing from 0.881 to 0.696. Given the clinical priority of identifying high-risk patients, this trade-off is acceptable, collectively demonstrating the robust predictive capability and generalizability of the XGBoost model under different data distribution conditions. Risk-stratified survival analysis based on the XGBoost model revealed a significant difference in DFS between the high-risk and low-risk groups (log-rank *p* < 0.0001). Patients in the low-risk group maintained consistently high and stable DFS rates throughout the follow-up period. In contrast, patients in the high-risk group showed early event accumulation, with DFS declining sharply around 18 months postoperatively—consistent with the first recurrence peak in breast cancer (26). These results demonstrate that the XGBoost model effectively stratifies recurrence and metastasis risk.

The SHAP analysis was applied to interpret the prediction logic of the optimal XGBoost model. The results identified SII, LVI, and TNM stage as the three most influential risk factors for adverse postoperative prognosis, whereas PNI was the most significant protective factor. SII is a composite index comprising neutrophils, lymphocytes, and platelets, which exerts critical effects on tumor progression via its components. Lymphocytes exert antitumor effects by promoting tumor cell apoptosis and inhibiting proliferation (27). Neutrophils form neutrophil extracellular traps that promote metastasis and remodel the tumor microenvironment in various solid tumors (28, 29). Platelets contribute to epithelial–mesenchymal transition, angiogenesis, and NF-κB pathway activation, thus accelerating tumor progression (29, 30). Elevated SII is thus associated with increased recurrence risk. Clinically, SII is derived from routine blood tests, providing a cost-effective and readily available biomarker for risk stratification. The importance of TNM stage and LVI further highlights the critical roles of tumor burden and vascular invasion in breast cancer prognosis. Advanced TNM stage is associated with high invasiveness, proliferation and treatment resistance, elevating recurrence risk (31, 32). LVI indicates that tumor cells have invaded blood vessels or lymphatic vessels, can spread rapidly, and form micrometastases in distant organs; this process is typically unaffected by local treatment and ultimately leads to distant recurrence (33, 34). Clinically, these factors remain fundamental to surgical planning and adjuvant therapy decisions. A high PNI reflects adequate nutritional reserves and intact immune function, enhancing resistance to tumor progression and metastasis while improving tolerance to antitumor treatments, such as radiotherapy, chemotherapy, and immunotherapy, thereby contributing to improved survival outcomes (35, 36). In clinical practice, patients with low PNI may benefit from preoperative nutritional support, and this index can serve as a modifiable target for risk reduction. Age also emerged as an important predictor, with younger age associated with increased risk. This finding aligns with prior studies demonstrating that younger breast cancer patients tend to present with more aggressive tumor characteristics, including higher histological grade and poorer hormone receptor status, and face higher mortality rates, with younger age identified as an independent risk factor for both disease-free and overall survival (37–40). Clinically, age-based risk stratification is critical, as younger patients may need more intensive surveillance and tailored adjuvant therapy. The SHAP interaction analysis revealed widespread but generally weak synergistic effects among features, with stronger interactions between TNM stage and LVI, and between age and TNM stage. These findings suggest that a high tumor burden combined with vascular invasion produces a synergistic effect on metastasis promotion, rather than a simple additive effect. Similarly, the interaction between advanced age and TNM stage may be related to immune senescence and age-associated microenvironmental changes, jointly accelerating disease progression. These findings enhance the model interpretability and confirm its capacity to capture complex nonlinear relationships among prognostic factors.

Based on these findings, the model may support several clinical applications: (1) early identification of high-risk patients, enabling optimized allocation of medical resources and focused follow-up and adjuvant treatment; (2) management of modifiable prognostic factors, such as systemic inflammation, and nutritional indicators such as SII and PNI are clinically modifiable and may represent targets for comprehensive intervention; and (3) individualization of follow-up strategies, allowing transition from uniform follow-up schedules to risk-stratified follow-up models.

This study has several limitations. First, the sample size of the TNBC subgroup was limited, which precluded reliable subgroup validation for this aggressive breast cancer subtype. Second, other potentially machine learning efficient models have not been explored. Third, certain important prognostic variables, such as preoperative medication history, were not included and should be incorporated into future studies to improve predictive performance. Fourth, the SHAP analysis indicated that features such as SII and PNI contributed most strongly to prediction, raising the possibility that the model may be overly sensitive to these variables while underutilizing other features that may be relevant to specific high-risk subgroups, which warrants further investigation using larger external datasets.

## 5 Conclusion

The XGBoost model constructed, using perioperative data from patients undergoing breast cancer surgery, outperformed the LR, RF, SVM, and GBM models in predicting adverse postoperative prognosis. The SHAP method can effectively facilitate the clinical application and translation of machine-learning technologies.

## Data Availability

The original contributions presented in the study are included in the article/Supplementary Material, further inquiries can be directed to the corresponding author.
